# The therapeutic effects of arginine on hyperglycemia, pancreatic α/β cells, oxidative stress, and bone turnover markers (CTx and OPG) in alloxan-induced diabetic rats

**DOI:** 10.3389/fendo.2026.1908992

**Published:** 2026-08-11

**Authors:** Fatimah A. Al-Saeed, Norah Hamdi, Sayed Soliman Abdel Ghfar, Montaser Elsayed Ali

**Affiliations:** 1 Department of Biology, College of Science, King Khalid University, Abha, Saudi Arabia; 2 Department of Animal Productions, Faculty of Agriculture, Al-Azhar University, Cairo, Egypt; 3 Department of Animal Productions, Faculty of Agriculture, Al-Azhar University, Assiut, Egypt

**Keywords:** alloxan, arginine, C-telopeptide (CTx), diabetes mellitus (DM), osteoprotegerin (OPG), oxidative stress, pancreatic α/β cells

## Abstract

This study evaluated the therapeutic effects of arginine (Arg) on hyperglycemia, pancreatic α/β cells integrity, oxidative stress biomarkers, C-telopeptide (CTx), and osteoprotegerin (OPG) in a rat model of alloxan-induced diabetes mellitus. A total of 36 male Wistar rats, weighing 126.14 ± 3.25 g and aged 75.47 ± 2.25 days (means ± SD), were randomly allocated into three equal group: control group (CG), diabetic control group (DCG), and arginine-supplemented diabetic group (Arg-DCG). Arg was administered orally at 400 mg/kg body weight daily for 28 days following diabetic confirmed. The DCG developed sustained hyperglycemia throughout the study period compared to the CG and Arg-DCG. Regarding oxidative stress, the Arg-DCG showed significant improvements in the antioxidant enzymes glutathione peroxidase (GPx), superoxide dismutase (SOD), and catalase (CAT) compared to the DCG. Conversely, malondialdehyde (MDA) a marker of lipid peroxidation, was significantly higher in the DCG relative to the CG, with only partial attenuation observed in the Arg-DCG. Western blot analysis showed a significant increase in relative CTx band intensity and a significant decrease in relative OPG band intensity in the Arg-DCG compared to DCG. Furthermore, qPCR analysis revealed that OPG gene expression (2^-ΔΔ Ct) was significantly higher in the Arg-DCG compared to the CG (2.03 vs 1.00), but remained lower than that of the DCG (2.03 vs 2.46). Histopathological analysis revealed significant improvements in pancreatic α/β cell repair in the Arg-DCG compared to the DCG, showcasing better-defined islets, reduced degeneration, and intact acinar tissue, suggesting partial protection from alloxan-induced damage. In conclusion, arginine therapy may effectively alleviate hyperglycemia, preserve pancreatic α/β cell integrity, and enhance antioxidant defense mechanisms in alloxan-induced diabetic rats.

## 1 Introduction

Diabetes mellitus (DM) is a chronic metabolic disorder characterized by hyperglycemia resulting from defective insulin secretion, impaired insulin action, or both, leading to disturbed metabolism of carbohydrates, proteins, and fats (1, 2). DM is associated with several complications, including vascular damage, retinopathy, nephropathy, and metabolic disturbances (3), which may eventually contribute to bone deterioration if left unmanaged (4). In experimental research, DM is commonly induced in rats to study its long-term complications. This is typically achieved via a single intraperitoneal (IP) or subcutaneous injection of alloxan at doses ranging from 70 to 200 mg/kg (5). Alloxan is widely used to induce DM in rats due to its ability to selectively destroy pancreatic β-cells (6). The diabetogenic effect of alloxan involves the generation of reactive oxygen species (ROS) and free radicals, as well as the oxidation of thiol groups in glucokinase, an essential enzyme for glucose metabolism (7–9). Diabetes indication is confirmed by elevated blood glucose concentrations > 250 mg/dL, polyuria, glucosuria, and significant weight loss, typically assessed 24 and/or 72 hours after alloxan injection (10). Alloxan-induced DM stimulates glutathione (GSH) oxidation (11).

Arginine (Arg) promotes polyamine synthesis necessary for cell proliferation and regeneration, suggesting β-cell regeneration and improving pancreatic tissue morphology (12). Furthermore, Arg may contribute to diabetes management by enhancing the L-arginine-nitric oxide (NO) pathway, which improves vascular regulation and insulin secretion (13), It increases NO production, resulting in better blood flow to the pancreas and potentially enhancing insulin delivery and β-cell function (14). Studies indicate that arginine supplementation in diabetic models reduces blood glucose levels, improves insulin sensitivity, and restores pancreatic arginase activity, further supporting enhanced endocrine function and β-cell regeneration (15). Furthermore, Arg restores pancreatic arginase activity, which is associated with enhanced endocrine function, suggesting the regeneration of pancreatic β-cell from ductal and acinar cells (16). However, NO modulates osteoblast activity and inhibits osteoclast-mediated bone resorption via NO-dependent pathways, potentially explaining the changes observed in OPG and CTx expression in the study (17). Oral L-arginine at 400 mg/kg for 28 days enhanced oxidative stress markers and gastrointestinal tissues in alloxan-induced diabetic rats, while significant changes in bone homeostasis markers were observed (18). This duration supports stable effects on glycemic control and biomarkers in preclinical diabetes research, aligning with animal welfare considerations.

Histopathological analysis have demonstrated that Arg supplementation reduces diabetes-induced pancreatic damage and promotes pancreatic cell regeneration (15). Additionally, Arg-supplementation increases NO production in osteoblasts and inhibits bone resorption by suppressing osteoclasts activity, thereby regulating bone remodeling (19). Indirectly, Arg enhances OPG activity and reduces bone resorption through NO-dependent pathways (20). In diabetic animals models, Arg-supplementation has been shown to increase OPG-mRNA expression in bone tissue, contributing to the prevention of osteoporosis by reducing bone resorption (21).

Although previous research has demonstrated beneficial effects of Arg supplementation in diabetic animal models (22–24), several knowledge gaps remain. Specifically, the concurrent effects of Arg on both pancreatic tissue integrity and bone turnover markers (CTx and OPG), and the integrity assessment of antioxidative stress markers alongside histopathological, remain limited. The theoretical hypothesis of the current study is that Arg exerts a protective and therapeutic effect against diabetes-associated pancreatic damage and bone metabolism disturbances. Therefore, this study aimed to evaluate the protective and therapeutic effect of Arg-supplementation on blood glucose levels, insulin sensitivity, and pancreatic α/β cells remodeling, as well as its ability to modulate the gene and protein expression of bone remodeling factors (OPG and CTx), and to assess its efficacy in reducing markers of oxidative stress in an alloxan-induced diabetic rat model.

## 2 Materials and methods

### 2.1 Chemicals

Alloxan monohydrate (A7413, CAS No. 2244-11-3, molecular formula C_4_H_2_N_2_O_4_·H_2_O, molecular weight 160.08 g/mol) was obtained from Sigma-Aldrich/Millipore Sigma (Merck KGaA, USA), with a purity of 98% as determined by high-performance liquid chromatography (HPLC). Arginine hydrochloride (C_6_H_14_N_4_O_2_·HCl, molecular weight 210.66 g/mol, 25 g, CAS No: 1119-34-2) was purchased from Tower CV of Hailing International E&T (Xi’an, Shaanxi, China), accompanied by a certificate of analysis (No: 25052701, production date May 27, 2025), which included the quality control results shown in **Table 1**.

**Table 1:** Certificate of analysis of arginine hydrochloride.

Item	Specification	Result	Test method
Appearance	White crystals or crystalline powder	Conforms	Visual
Assay	98.5 ~ 101.5%	99.3%	USP<541>
Residue on Ignition	≤ 0.1%	0.03%	USP<281>
Sulfate (SO_4_)	≤ 0.03%	< 0.03%	USP<221>
Chromatographic Purity	Individual impurity ≤ 0.5%; Total impurities ≤ 2.0%	Conforms	USP<621>
Specific Rotation [α]_20_^20^	+21.4° ~ +23.6°	+22.4°	USP<781S>
Loss on Drying	≤ 0.2%	0.17%	USP<731>
Chloride (Cl)	16.5 ~ 17.1%	16.8%	USP<541>

Performed by Tower CV of Hailing International E&T, Xi’an, Shaanxi, China.

### 2.2 Alloxan-induced diabetes

Diabetes mellitus (DM) was induced by a single intraperitoneally (IP) injection of alloxan monohydrate (Sigma-Aldrich/Millipore Sigma, USA) at a dose of 120 mg/kg of body weight. The alloxan was prepared in a solution by dissolving the crystallin powder in cold citrate buffer (0 – 4 °C; 0.1 M, pH4.5), protected from light to avoid photodegradation (25). Prior to alloxan administration, animals underwent 18 hours of fasting, ensuring consistent glycemic conditions and enhancing pancreatic β-cell susceptibility. An IP injection was carefully administered into the lower right abdomen using a 26-gauge needle, with 70% ethanol used before and after to prevent infection (26, 27). After alloxan injection, animals were provided with a 5% glucose solution in their drinking water for 24 hours to prevent severe hypoglycemia caused by excessive insulin from damaged β-cells. The solution was made by dissolving 50 g of D-glucose in 1 L of distilled water, after which it was replaced with regular drinking water. To confirm diabetes induction in rats, fasting blood glucose levels were measured 72 hours after alloxan administration, with levels > 250 mg/dL indicating diabetes (28). Blood glucose measurements were conducted on days 7, 14, and 21 after induction to assess diabetes stability (**Table 2**).

**Table 2:** Primer sequences for osteoprotegerin (OPG) gene amplification.

Genes	Primer sequence (5’ → 3’)	Primer Tm (°C)	GC (%)	Self-complementarity	Self-3’ complementarity
Osteoprotegerin (OPG)	For: TGTGGAATAGATGTCACCCTGTGC	62.32	60.00	4.00	2.00
	Rev: CACAGAGGTCAATGTCTTGGATGATC	59.67	60.00	2.00	1.00

### 2.3 Animals and grouping

A total of 36 male Wistar rats (R*attus norvegicus*), weighing 126.14 ± 3.25 g and aged 75.47 ± 2.25 days (means ± SD), were included in this study. Animals were obtained from Animal House, Faculty of Science, Al-Azhar University, Cairo, Egypt. All animals were housed in identical conditions (22 ± 3 °C, 65% humidity, 12-hour light/dark cycle) and fed a standard diet ad libitum. They underwent a 7-day acclimatization period to adapt to the laboratory conditions and minimize stress-related physiological variations. The animals were divided into three equal groups (n = 12 per group) using a computer-generated random number table, with body weight to minimize inter-group weight variation. This involved sorting them in ascending order and assigning them to blocks of three with similar weights (± 3.25 g):

- Control rats (CG): Healthy rats receiving no treatment and no disease induced.
- Diabetic control rats (DC0G): Rats receiving a single IP injection of alloxan (120 mg/kg of body weight).
- ARG-treated diabetic group (Arg-DCG): Rats receiving the same alloxan IP injection as DCG, followed by oral gavage of 400 mg/kg body weight of arginine hydrochloride dissolved in 0.5 mL saline after diabetes confirmation, the Arg dose of 400 mg/kg body weight was chosen according to Ezima et al. (18).

Individually, the animals were housed individually in stainless steel cages (30 x 20 x 15 cm) with a hard sawdust-covered surface, under standard laboratory conditions (22 ± 3 °C, 65% humidity, and 12-hour light/dark cycle) and fed a standard laboratory diet ad libitum. Cages were clearly labelled with rectangular white tags. All animals were monitored daily for health status, clinical signs, and data recording.

### 2.4 Biochemical analyses

Blood samples were collected from the ophthalmic plexuses using capillary tubes at days 0, 7, 14, and 21. All samples collection were performed after an overnight fasting period of 10 hours, with free access to water, to ensure standardization of glycemic measurements (29). After centrifugation (3000 rpm for 20 minutes), plasma was separated and stored at -20 °C for subsequent assays. Glucose concentration was measured via a GOD-PAP enzymatic colorimetric assay (Vitro Scient) (30). Insulin concentrations were assessed with an ELISA kits with an assay sensitivity of 1.467 μIU/ml (31). Glutathione peroxidase (GPx), superoxide dismutase (SOD), catalase (CAT), and malondialdehyde (MDA) were measured using colorimetric kinetic methods with kit from Bio-Diagnostic company (32, 33).

### 2.5 Histopathology

At the end of the experiment, euthanasia was performed by cervical dislocation under deep anesthesia. Prior to euthanasia, the animals were anesthetized intraperitoneally with ketamine (40 mg/kg) and xylazine (5 mg/kg). Death was confirmed by the cessation of cardiac and respiratory activity. For pancreatic histopathological examination, five rats from each group (CG, DCG, and Arg-DCG) were randomly selected and pancreatic tissue was collected.

Samples were fixed in 10% neutral buffered formalin, dehydrated through graded alcohol concentrations, cleaned in xylene, and embedded in paraffin wax. Sections of 5 μm thickness were cut, stained with hematoxylin and eosin (H&E), and examined under a light microscope (model number Luxeo 4D, Labomed, Inc., El Segundo, California, USA) (34). Histopathological evaluations were performed by an experienced pathologist blinded to the treatment group. Pancreatic islets were assessed for morphological features such as size, shape, vacuolation, cellular degeneration, inflammatory infiltration, and surrounding acinar integrity. H&E staining evaluates islet architecture but does not differentiate between α-cells and β-cells. Descriptions of cellular populations indicate general density and distribution patterns, not specific cell type identification.

### 2.6 Western blot analysis of CTx and OPG protein expression

CTx and OPG protein expression were analyzed in plasma samples collected from the ophthalmic plexus using Western blot technique. Samples were thawed on ice and diluted 1:5 in radioimmunoprecipitation assay (RIPA) lysis buffer containing 50 mM Tris-HCl (pH 7.4), 150 mM NaCl, 1% NP-40, 0.5% sodium deoxycholate, and 0.1% sodium dodecyl sulfate (SDS), supplemented with a protease inhibitor cocktail (cOmplete™ Mini, Roche, Germany). Total protein concentration was determined using the Bradford method (35), with bovine serum albumin (BSA) as a standard. Equal amounts of protein (40 µg per lane) were separated by 12% SDS polyacrylamide gel electrophoresis (PAGE), and subsequently transferred onto polyvinylidene difluoride (PVDF) membranes (Millipore, USA) using a semi-dry transfer system (Bio-Rad, USA). To ensure accurate measurement and adjust for inter-lane differences in protein loading and transfer efficiency, two complimentary normalization procedures were used: Ponceau S staining was performed on membranes after protein transfer using a 0.1% solution (Sigma-Aldrich, USA) in 5% acetic acid for 5 minutes. After rinsing with distilled water, images were captured. This total protein staining served as a reliable loading control for normalization, particularly for plasma samples that do not exhibit consistent housekeeping gene expression. Densitometric analysis was conducted to obtain the integrated optical density (IOD) for normalizing target protein band intensity. β-Actin reprobing involved stripping membranes with Restore™ Western Blot Stripping Buffer (Thermo Scientific, USA) for 15 minutes at room temperature, followed by washing with TBST and reprobing using mouse anti-rat β-actin monoclonal antibody (1:5000; Sigma-Aldrich, USA) to confirm consistent protein loading across all lanes. To prevent non-specific binding, membranes were blocked with TBST (Tris-buffered saline containing 0.1% Tween-20) for 1 hour at room temperature, then incubated overnight at 4 °C with rabbit anti-rat primary antibodies against CTx (1:500; Santa Cruz Biotechnology, USA) and OPG (1:1000; Abcam, UK). After three washes with TBST (1:10, 000; Cell Signaling Technology, USA), membranes were incubated with HRP-conjugated goat anti-rabbit secondary antibody for 1 hour at room temperature. Protein bands were visualized with an enhanced chemiluminescence substrate (SuperSignal™ West Pico PLUS, Thermo Scientific, USA) and documented using a ChemiDoc™ MP (Bio-Rad, USA) Imaging System. Band intensities were quantified with TotalLab software (Version 1.0.1; Nonlinear Dynamics, Newcastle, UK (www.totallab.com). Each Western blot staining experiment was conducted thrice with separate plasma samples from 12 mice per group.

### 2.7 Quantitative real-time polymerase chain reaction for gene expression analysis

#### 2.7.1 RNA isolate and quality assessment

Total RNA was purifilrd from whole blood samples obtained from all samples in each group (n = 12/groups) using the TRIzol™ Reagent (15596026, Life Technologies, USA), was applied for total RNA purification from blood samples according to manufacturer protocol. TRIzol™ Reagent is a monophasic solution of phenol, guanidine isothiocyanate, and other proprietary components which facilitate the isolation of a variety of RNA species of large or small molecular size. TRIzol™ Reagent allows performing sequential precipitation of RNA, DNA, and proteins from a single sample. Blood samples were prepared by transferring to a centrifuge tube with RBC Lysis Buffer and incubating for 10 minutes. Cells were pelleted by centrifugation and resuspended for further processing. For RNA isolation, 1, 200 µL of TRIzol™ Reagent was added to each tube to resuspend the cells, followed by chloroform (0.2 mL per 1 mL of TRIzol™ Reagent), incubation, and centrifugation for 2–3 minutes, then centrifuged at 12, 000 × g for 15 minutes at 4 °C, which separated the mixture into three phases. The aqueous phase containing RNA was collected, and RNA was precipitated with isopropanol, washed with 75% ethanol, and air-dried for 5–10 minutes, then dissolved in 20–50 µL of RNase-free water by pipetting up and down, followed by incubation in a heat block at 55–60 °C for 10–15 minutes. Quality control involved spectrophotometric assessment of RNA concentration and purity, using A260/280nm ratios to ensure high purity before cDNA synthesis.

#### 2.7.2 Primer design

Primer sequences for OPG, β-actin were designed based on sequences as shown in **Table 2**. The OPG primer combination (forward: 5′-TGTGGAATAGATGTCACCCTGTGC-3′; reverse: 5′-CACAGAGGTCAATGTCTTGGATGATC-3′) was constructed using the Rattus norvegicus OPG gene sequence, which has an amplicon length of 92 bp and a melting temperature (Tm) of around 60 °C. BLAST analysis against the Rattus norvegicus reference genome validated primer specificity, ensuring no off-target amplification.

#### 2.7.3 cDNA synthesis

Complementary DNA (cDNA) synthesis was performed using 1 µg of total RNA and the QuantiTect Reverse Transcription Kit. A gDNA Wipeout Buffer eliminated contaminating genomic DNA without DNase treatment. The genomic DNA elimination involved 2 µL of buffer, template RNA, and RNase-free water, incubated at 42 °C for 2 minutes. Subsequently, a reverse-transcription master mix was prepared, and the template RNA was added, followed by incubation at 42 °C for 15 minutes and 95 °C for 3 minutes to inactivate the enzyme. The cDNA was stored at –20 °C for later qPCR analysis, including a reverse transcription-negative control to check for genomic DNA contamination.

#### 2.7.4 qPCR assay and technical validation

Quantitative real-time PCR was conducted using the Rotor-Gene Q system (Qiagen, USA) with SYBR^®^ Green chemistry. Each 25 µL reaction included 12.5 µL of Master Mix (2×; Thermo Scientific, USA), 300 nM forward and reverse primers, and 30 ng of cDNA. The thermal cycling involved an initial denaturation at 95 °C for 10 minutes, followed by 45 cycles of 95 °C for 10 seconds, 60 °C for 15 seconds, and 72 °C for 15 seconds. Melt curve analysis was performed to assess amplicon specificity, and no-template controls were included to check for contamination. The validation of qPCR results included several steps to ensure data reliability and accuracy. Primer optimization involved testing concentrations (100–600 nM) and annealing temperatures (55–65 °C), determining optimal conditions at 300 nM and 60 °C. PCR efficiency was assessed with standard curves, yielding efficiencies of 98.2% for OPG and 99.1% for β-actin, both within the acceptable range of 90–110%. Melt curve analysis showed distinct peaks confirming product specificity. Agarose gel electrophoresis confirmed the presence of expected product sizes (OPG: 92 bp; β-actin: 103 bp). Quality control standards included a Cq standard deviation of less than 0.25 cycles between duplicates, no amplification in NTC wells, and a β-actin Cq variation of less than 1.0 cycles across samples.

#### 2.7.5 Relative gene expression analysis

The OPG gene’s relative expression was determined using the 2^–ΔΔCt method, normalizing threshold cycle (Ct) values to the housekeeping gene β-actin. The control group served as the calibrator with a ΔΔCt value of 0, indicating a fold change of 1.00. The equations used were ΔCt (tested) = Ct (target in tested group) – Ct (reference in tested group), ΔCt (calibrator) = Ct (target in control group) – Ct (reference in control group), ΔΔCt = ΔCt (tested) – ΔCt (calibrator), and Fold Change = 2^–ΔΔCt (36).

### 2.8 Statistical analysis

Statistical analyses were conducted using SPSS version 26 (IBM Corp., Armonk, NY, USA). The Kolmogorov-Smirnov test was used to assess data normality and Levene’s test evaluating variance homogeneity. One-way ANOVA was used to compared experimental groups (CG, DCG, Arg-DCG), and Tukey’s HSD *post-hoc* test was applied when significant effects were noted (P < 0.05). The generalized linear model used was Yij = μ + Ti + Eij, where Yij is the observation, μ is the overall mean, Ti is the group effect, and Eij denotes the experimental error.

## 3 Results

### 3.1 Glucose, insulin, and histopathology

The data presented in **Table 3** reported that no significant (P > 0.05) differences were observed in blood glucose and insulin concentrations among groups at day 0, indicating a consistent and comparable starting point. Furthermore, the DCG developed sustained hyperglycemia, with blood glucose concentrations (P < 0.01) elevated to 398.33, 378.50, and 452.83 mg/dL on days 7, 14, and 21, respectively, compared to the CG and Arg-DCG. In contrast, the Arg-DCG maintained normoglycemic levels (156.33, 153.01, and 144.50 mg/dL on days 7, 14, and 21, respectively), that were not statistically different (P > 0.05) from those of the CG group. Additionally, the DCG suffered a sustained insulin deficiency (P < 0.01) compared to the CG and Arg-DCG. In contrast, insulin levels in the Arg-DCG were maintained at near-CG concentrations.

**Table 3:** Blood glucose and insulin concentrations in control and experimental groups.

Diabetic parameters		CG	DCG	Arg-DCG	SEM	P-Value
Glucose Concentration (mg/dL)	At 0 days	137.00 ns	135.33 ns	136.67 ns	0.94	0.768
	At 7 days	140.67 b	398.33 a	156.33 b	29.39	0.001
	At 14 days	138.17 b	378.50 a	153.01 b	27.36	0.001
	At 21 days	135.50 b	452.83 a	144.50 b	36.46	0.001
Insulin Concentrations (uIU/ml.)	At 0 days	18.21 ns	17.81 ns	17.95 ns	0.18	0.662
	At 7 days	19.92 a	3.17 b	20.41 a	2.08	0.001
	At 14 days	21.61 a	3.31 b	19.13 a	2.01	0.001
	At 21 days	19.42 a	3.15 b	19.05 a	1.85	0.001

Different superscript letters (a, b, c) within a row denote statistically significant differences according to Tukey’s HSD multiple range test (P < 0.001). SEM, Standard Error of the Mean; CG, Normal control; DCG, Diabetic control; Arg-DCG, Diabetic + Arginine treated.

**Figure 1:**
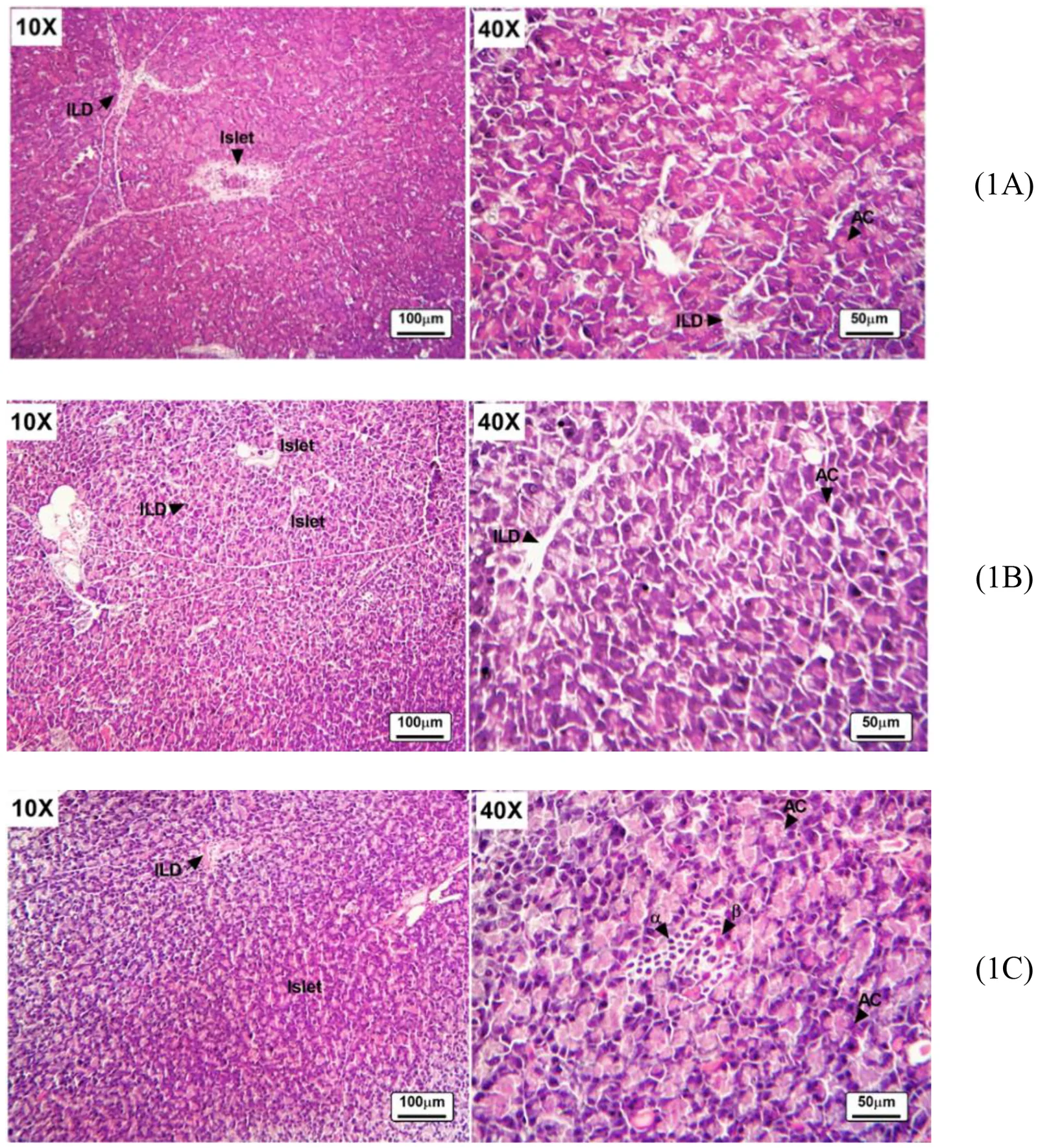
Photomicrographs of pancreatic sections stained with H&E. The CG group **(A)** shows normal islet architecture, acini, and intralobular ducts. The DCG group **(B)** displays irregular islet sizes, extensive vacuolation, cellular disintegration, abnormal nuclei, tubular fibrosis, and exocrine tissue degeneration. The Arg-DCG group **(C)** indicates moderate improvement, with less distortion of islets, reduced cellular degeneration, intact acinar tissue, and increased cellular density.

Histopathological examination of H&E-stainepancreatic sections in the CG showed normal exocrine tissue The islets were round to oval, with granulated cytoplasm and densely packed cells, showing no degeneration or inflammatory infiltration, and acinar cells appeared normal with preserved architecture (**Figure 1A**). The DCG showed significant histopathological damage, with irregularity in islet size, extensive vacuolation, cellular disintegration in the peripheral regions of the islets (areas typically occupied by α-cells), and abnormal nuclei in the central regions (areas typically occupied by β-cells), tubular fibrosis, and degeneration of adjacent exocrine tissue (**Figure 1B**). In contrast, the Arg-DCG showed moderate morphological improvement with identifiable islets exhibiting less distortion and reduced cellular degeneration. Acinar tissue remained intact with minimal inflammatory infiltration, and there was an increase in overall cellular density within the islets (**Figure 1C**).

### 3.2 Complete blood counts

The Arg-DCG showed a significant increase (P < 0.01) in red blood cell (RBC) count, hemoglobin (Hb/Hgb), hematocrit (Hct), mean corpuscular volume (MCV), and mean corpuscular hemoglobin (MCH), compared to the DCG, while no statistically significant differences were observed compared to the CG (**Table 4**). Furthermore, there was a significant decrease (P < 0.01) in white blood cell (WBC) and platelet (PLT) counts in the DCG compared to the CG, with values in the Arg-DCG approaching those of the CG (P > 0.05). The neutrophil count decreased significantly (P < 0.05) in the DCG compared to the CG. No significant differences in lymphocyte or monocyte percentages were found among the groups (P > 0.05). However, the total leukocyte count (TLC) was significantly higher in the DCG compared to the CG and Arg-DCG (P < 0.05), while mean corpuscular hemoglobin concentration (MCHC) showed no significant differences (**Table 4**).

**Table 4:** Complete blood count (CBC) parameters in control and experimental groups.

Group	CG	DCG	Arg-DCG	SEM	P-Value
RBC (×10^6^/μL)	6.73 a	6.05 b	6.68 a	0.13	0.017
Hb/Hgb (g/dL)	14.6 a	9.00 b	14.37 a	0.93	0.001
Hct (%)	44.8 a	32.77 b	40.77 a	1.89	0.002
MCV (fL)	66.57 a	42.71 b	61.44 a	3.73	0.001
MCH (pg)	21.69 a	18.49 b	21.56 a	0.58	0.006
MCHC (g/dL)	32.59 ns	36.48 ns	36.01 ns	0.81	0.080
WBC (×10³/μL)	24.9 a	15.72 b	18.35 ab	1.66	0.035
PLT (×10³/μL)	757 a	387 b	516.67 ab	64.63	0.026
TLC (×10³/μL)	17.43 b	21.42 a	15.92 b	0.96	0.019
Neutrophils (%)	0.20 a	0.10 b	0.14 ab	0.02	0.044
Lymphocytes (%)	0.72 ns	0.74 ns	0.73 ns	0.01	0.796
Monocytes (%)	0.56 ns	0.16 ns	0.31 ns	0.08	0.094

Different superscript letters (a, b, c) within a row denote statistically significant differences according to Tukey’s HSD multiple range test (P < 0.001). SEM, Standard Error of the Mean; CG, Normal control; DCG, Diabetic control; Arg-DCG, Diabetic + Arginine treated. RBC, Red Blood Cells; Hb/Hgb, Hemoglobin; Hct, Hematocrit; MCV, Mean Corpuscular Volume; MCH, Mean Corpuscular Hemoglobin; MCHC, Mean Corpuscular Hemoglobin Concentration; WBC, White Blood Cells; PLT, Platelets.

### 3.3 Oxidative marker

The key antioxidant enzymes showed a significance (P < 0.01) decrease in glutathione peroxidase (GPx), superoxide dismutase (SOD), and catalase (CAT) in the DCG compared to the CG. In contrast, the Arg-DCG exhibited a significant improvement (P < 0.01) in GPx, SOD, and CAT levels compared to the DCG. Regarding lipid peroxidation, malondialdehyde (MDA) levels, a marker of the oxidative damage were significantly higher (P < 0.001) in the DCG compared to the CG, with significantly lower levels observed in the Arg-DCG compared to the DCG, although they remained higher than the CG (**Table 5**).

**Table 5:** The assessment evaluates the effectiveness of arginine therapy on oxidative markers including; Glutathione Peroxidase (GPx), Superoxide Dismutase (SOD), catalase (CAT), and malondialdehyde (MDA).

Group	CG	DCG	Arg-DCG	SEM	P-Value
CTx	36.59 a	17.29 c	21.94 b	1.60	<0.001
SOD	27.38 a	18.94 b	27.63 a	0.77	<0.001
CAT	97.2 6a	38.24 c	42.56 b	5.24	<0.001
MDA	0.87 c	2.33 a	1.44 b	0.12	<0.001

Different superscript letters (a, b, c) within a row denote statistically significant differences according to Tukey’s HSD multiple range test (P < 0.001). SEM, Standard Error of the Mean; CG, Normal control; DCG, Diabetic control; Arg-DCG, Diabetic + Arginine treated.

### 3.4 C-Telopeptide protein expression

**Figure 2:**
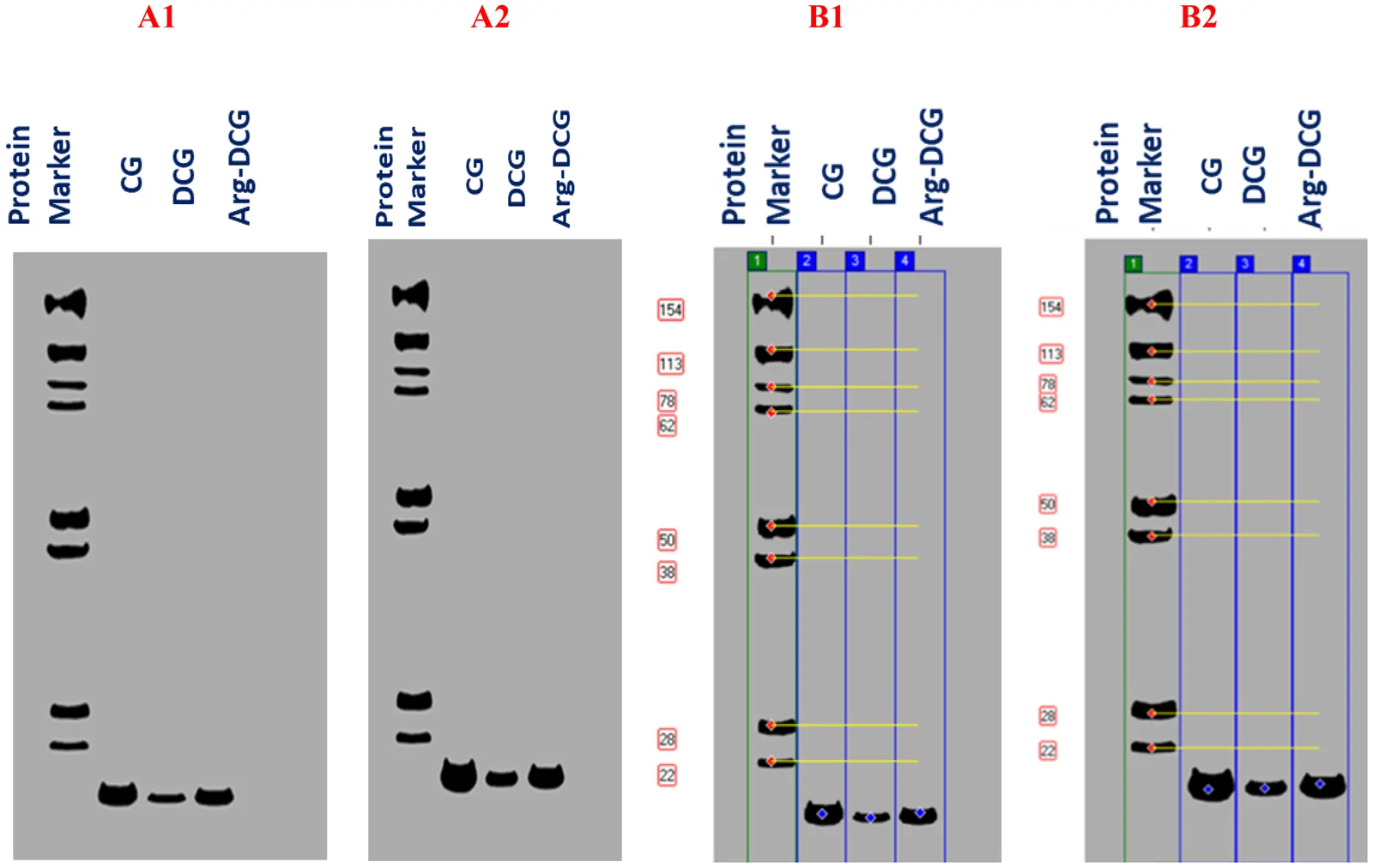
Western blot analysis of C-telopeptide (CTx) protein expression. Representative immunoblots illustrating CTx protein bands across the experimental groups. Panel displays the determination of protein molecular weight using standard protein markers. Panel shows CTx isoform signals detected in two separate sets of samples, representing larger (**A2** and **B2**) and smaller (**A1** and **B1**) loading series, respectively, to confirm specificity and linearity of the immunodetection.

Western blot analysis of plasma samples revealed differential in CTx protein expression levels among the experimental groups (**Figure 2**). The CG exhibited the highest CTx protein band intensity, whereas the DCG showed the lowest band intensity. The Arg-DCG displayed an intermediate band intensity between CG and DCG. Furthermore, densitometric analysis in **Table 6** confirmed a significant decrease in relative CTx band intensity in the DCG compared to the CG and Arg-DCG. Notably, the Arg-DCG showed a significant increase in CTx band intensity expression relative to the DCG, although it remained lower than that of the CG.

**Table 6:** Densitometric analysis of CTx and OPG protein expression band intensities expressed as relative arbitrary units.

Group	CG	DCG	Arg-DCG	SEM	P-Value
CTx	17.25 a	4.48 c	12.28 b	0.90	<0.001
OPG	2.33 c	14.11 a	7.59 b	0.84	<0.001

Different superscript letters (a, b, c) within a row denote statistically significant differences according to Tukey’s HSD multiple range test (P < 0.001). SEM, Standard Error of the Mean; CG, Normal control; DCG, Diabetic control; Arg-DCG, Diabetic + Arginine treated.

### 3.5 Osteoprotegerin protein expression

**Figure 3:**
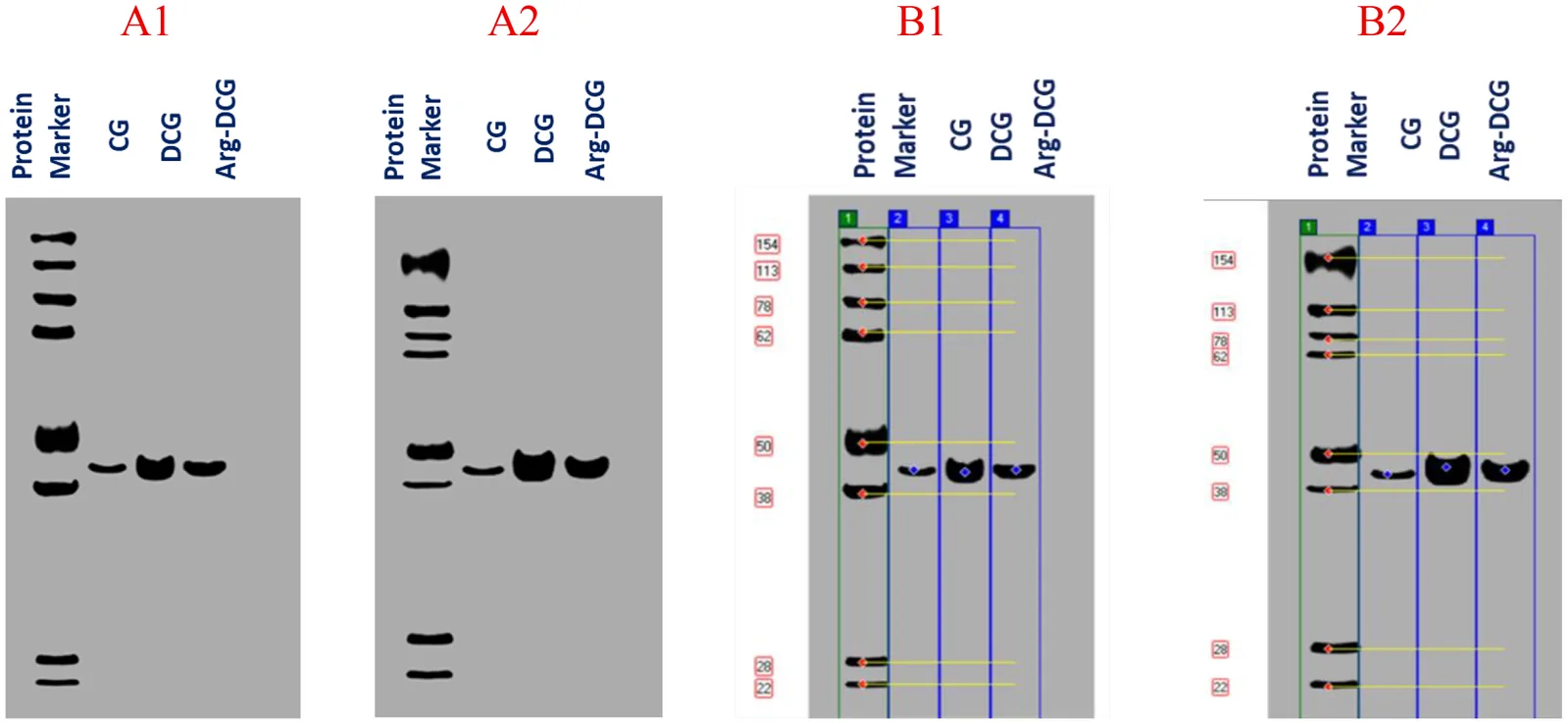
Western blot analysis of osteoprotegerin (OPG) protein expression. Representative immunoblots illustrating OPG protein bands across the experimental groups. Panel displays the determination of protein molecular weight using standard protein markers. Panel shows OPG isoform signals detected in two separate sets of samples, representing larger (**A2** and **B2**) and smaller (**A1** and **B1**) loading series, respectively, to confirm specificity and linearity of the immunodetection.

Western blot analysis of plasma samples revealed differential in OPG protein expression levels among the experimental groups (**Figure 3**). The CG exhibited the lowest OPG protein band intensity, whereas the DCG showed the highest band intensity. The Arg-DCG exhibited an intermediate band intensity between CG and DCG. Furthermore, densitometric analysis in **Table 6** confirmed a significant increase in relative OPG band intensity in the DCG compared to the CG and Arg-DCG. Notably, the Arg-DCG showed a significant decrease in band intensity expression relative to the DCG, although it remained higher than that of the CG.

### 3.6 Osteoprotegerin gene expression

OPG mRNA expression levels in plasma samples were quantified using qPCR with β-actin as the housekeeping gene (**Table 7**). Relative gene expression was calculated using the 2^-^ΔΔCt method. The ΔCt value in the CG was 2.50, which served as the calibration baseline. The DCG showed a significant increase (P < 0.01) in OPG gene expression, with a fold change of 2.46 compared to the CG. The Arg-DCG also exhibited a significant increase (P < 0.01) relative to the CG, with a fold change of 2.03. Notably, the Arg-DCG showed a lower fold change compared to the DCG. Furthermore, qPCR fold change values (2^−^ΔΔCt) indicate logarithmic amplification of mRNA transcripts and differ in units from Western blot analysis results. Consequently, protein and gene expression data were analyzed separately.

**Table 7:** Relative expression of osteoprotegerin (OPG) gene normalized to β-actin using the 2^(-ΔΔCt) method.

group	CG	DCG	Arg-DCG	SEM	P-Value
Mean Ct (OPG)	31.83	31.16	30.80	0.01	<0.001
Mean Ct (β-actin)	29.33	29.96	29.33	0.01	<0.001
Mean ΔCt (Ct-OPG – Ct-β-actin)	2.50	1.20	1.47	0.11	<0.001
ΔΔCt (ΔCt Sample – ΔCt CG)	0.00	-1.30	-1.03	–	–
Fold Change (2^(-ΔΔCt))	1.00	2.46	2.03	0.38	<0.001

Data are presented as means. CG, Control group; DCG, Diabetic control group; Arg-DCG, Arginine-treated diabetic group; Ct, Threshold cycle; ΔCt, Delta Ct; ΔΔCt, Delta-Delta Ct. Fold change values > 1 indicate upregulation relative to the CG. SEM, Standard Error of the Mean; CG, Normal control; DCG, Diabetic control; Arg-DCG, Diabetic + Argininetreated.

## 4 Discussion

The presented study evaluated the therapeutic effects of the arginine (Arg) supplementation on hyperglycemia, pancreatic α/β cells, oxidative stress biomarkers, and bone turnover markers (CTx and OPG) in an alloxan-induced diabetic rats. The main finding demonstrated that Arg therapy significantly attenuated hyperglycemia, preserved insulin levels, restore antioxidant enzyme activity, improves pancreatic histology, and modulated CTx and OPG expression. These results supported the hypothesis that Arg exerts protective effect against diabetes-associated pancreatic damage and bone metabolism disturbances. The 400 mg/kg body weight dose of arginine was selected based on literature review, showing validation in rodent models for diabetes and oxidative stress (18). This dosage significantly improved glucose tolerance and falls within the common preclinical range of 200–800 mg/kg, balancing efficacy and safety (37, 38).

### 4.1 Arginine and hyperglycemia

Arginine acts as a substrate for the nitric oxide (NO) synthase, enhancing NO production (13). According to Musumeci et al. (39), NO availability via Arg regulates blood glucose levels and improves tissue functions. In this study, the DCG developed sustained hyperglycemia throughout the experiment, while the Arg-DCG maintained normoglycemic compared to the CG. This finding is consistent with El-Alfy et al. (40), who reported that alloxan-induced diabetic animals exhibit persistent hyperglycemia accompanied by oxidative stress, and with Kocher and Umathe (10), who demonstrated that the Arg treatment prevents glucose elevation and restores normal levels (41). Furthermore, insulin levels in the Arg-DCG remained near those of the CG.

### 4.2 Complete blood counts

The results showed that the DCG suffered from severe deficiencies in RBC count, Hb, and Hct, reflecting a state of diabetic anemia. The Arg-DCG showed a significant improvement in these values, bringing them similar to the control group. Evidence suggests that treating diabetic mice with arginine derivatives improves the functional and morphological status of red blood cells, partly due to its hypoglycemic effect, which improves the bone marrow environment (42). Furthermore, the study revealed a notable increase in PLT in the arginine-treated group versus the untreated cohort, supporting prior research that indicates L-arginine may lower elevated platelet counts in diabetes, thereby offering protection against thrombotic issues (43). This statement highlights arginine’s role in mitigating oxidative stress and chronic inflammation via its influence on the nitric oxide pathway, which in turn normalizes immune cell counts.

### 4.3 Pancreatic histopathology

Histopathological examination revealed that Arg-DCG rats showed moderate morphological improvement, with identifiable islets exhibiting less distortion, reduced cellular degeneration, intact acinar tissue, and intact acinar tissue, and increase in the peripheral regions of the islets density. Alloxan-induced diabetic is known to a decrease polyamines levels and reduce pancreatic arginase activity (44). Arg supplementation has been shown to restore arginase activity in damaged pancreatic tissues (44). The Arg-NO pathway, along with polyamines, plays a protective role in β-cell function and promotes regeneration from ductal and acinar cells (15).

### 4.4 Oxidative stress

Oxidative stress is a key contributor to diabetes complications (45, 46). In the current study, the DCG showed significantly reduced activities of GPx, SOD, and CAT, while Arg-DCG showed marked improvement in these antioxidant enzymes. Similarly, Li et al. (47) reported that the L-Arg treatment restored GSH-px and CAT mRNA expression in a high glucose-induced oxidative stress model. Additionally, Arg supplementation has been shown to increase SOD activity and expression in rats, enhancing antioxidant capacity (48–50). MDA, a marker of lipid peroxides, was significantly elevated in DCG, with partial attenuation in Arg-DCG. These results align with previous studies demonstrating Arg’s capacity to reduce oxidative stress by lowering MDA levels (51, 52).

### 4.5 CTx and OPG expression

Western blot examination showed that CTx protein expression was highest in the CG, lowest in the DCG, and intermediate in the Arg-DCG. In contrast, OPG expression was lowest in the CG and highest in the DCG, with intermediate levels in the Arg-DCG. These findings support the role of arginine in regulating bone metabolism via the L-arginine-NO pathway (13). In diabetic rats, arginine supplementation lowers serum CTx levels, indicating reduced bone resorption, and increases OPG mRNA expression. Nitric oxide, generated from arginine, activates pathways in osteoblasts that enhance OPG production (21, 53). Reduced NO availability due to oxidative stress leads to decreased OPG and increased osteoclast activity, while arginine supplementation restores NO and improves OPG/RANKL ratios, consequently affecting CTx and OPG protein levels. A notable increase in OPG gene expression in supplemented diabetic rats supports the transcriptional effect of arginine on bone metabolism (54). Elevated OPG decreases osteoclast differentiation, which reduces bone resorption. This study evaluated CTx and OPG expression in plasma samples to determine systemic levels of these bone turnover indicators. The observed pattern suggests that Arg supplementation positively modifies these markers. Future studies should look at bone mineral density and histomorphometry characteristics to determine the functional implications of these alterations (21).

### 4.6 Study limitations

While this study provides important insights into the therapeutic potential of L-arginine in the management of diabetes, it is essential to consider the following methodological limitations. The study included three experimental groups (CG, DCG, and Arg-DCG), and lacked an arginine-only treatment group, which may hinder the evaluation of arginine’s effects. However, the marked differences between the Arg-DCG and DCG, along with the normal blood glucose and insulin levels in the CG, suggest that the observed therapeutic effects are attributable to arginine. Future research should include additional control groups to validate the specific effects of arginine. Arginine supplementation has been shown to benefit blood sugar levels, pancreatic health, and oxidative stress. However, the study’s limitation lies in not exploring pathways like nitric oxide (NO) signaling and Nrf2-mediated antioxidant activation. Future research should focus on these pathways. Furthermore, the 28-day treatment period for L-arginine was selected based on preclinical evidence. Although studies have varied in duration, the ideal length for L-arginine supplementation in diabetic models is still uncertain. Future research should investigate longer treatment periods (8–12 weeks) to assess the lasting effects on glycemic control. A limitation of this study is the lack of histological analysis of bone tissue. Although significant changes in bone turnover markers (CTx and OPG) have been observed, these provide only indirect evidence of bone metabolism. Future research should include bone histomorphometry to validate these changes and confirm the effects of arginine on bone remodeling.

## 5 Conclusion

Arginine supplementation attenuates hyperglycemia, preserves pancreatic α/β cell integrity, improves antioxidant enzyme activation (GPx, SOD, and CAT), and modulates the expression of bone turnover markers (CTx and OPG) in alloxan-induced diabetic rats. These data indicate that Arg may serve as a potential adjuvant therapy for diabetes mellitus and its associated bone complications. Future research should prioritize a structured plan to explore the L-arginine-nitric oxide pathway in pancreatic tissue, necessitating long-term toxicity and efficacy studies of at least 8 weeks. Additionally, assessments of functional bone health, particularly long-term bone mineral density, are vital for evaluating the impact on skeletal integrity.

## Data Availability

The raw data supporting the conclusions of this article will be made available by the authors, without undue reservation.
